# Construction and Application of a Non-Enzyme Hydrogen Peroxide Electrochemical Sensor Based on Eucalyptus Porous Carbon

**DOI:** 10.3390/s18103464

**Published:** 2018-10-15

**Authors:** Shuisheng Wu, Nianyuan Tan, Donghui Lan, Chak-Tong Au, Bing Yi

**Affiliations:** Hunan Provincial Key Laboratory of Environmental Catalysis & Waste Recycling, College of Chemistry and Chemical Engineering, Hunan Institute of Engineering, Xiangtan 411104, China; tanny2011@126.com (N.T.); donghuilan@hnu.edu.cn (D.L.); pctau@hkbu.edu.hk (C.-T.A.)

**Keywords:** biomorphic porous carbon, electrochemical sensor, hydrogen peroxide, electrocatalytic properties, characterization

## Abstract

Natural eucalyptus biomorphic porous carbon (EPC) materials with unidirectional ordered pores have been successfully prepared by carbonization in an inert atmosphere. X-ray diffraction (XRD), Fourier transform infrared spectroscopy (FT-IR) and scanning electron microscope (SEM) were employed to characterize the phase identification, microstructure and morphology analysis. The carbon materials were used to fabricate electrochemical sensors to detect hydrogen peroxide (H_2_O_2_) without any assistance of enzymes because of their satisfying electrocatalytic properties. It was immobilized on a glassy carbon electrode (GCE) with chitosan (CHIT) to fabricate a new kind of electrochemical sensor, EPC/CHIT/GCE, which showed excellent electrocatalytic activity in the reduction of H_2_O_2_. Meanwhile, EPC could also promote electron transfer with the help of hydroquinone. The simple and low-cost electrochemical sensor exhibited high sensitivity, and good operational and long-term stability.

## 1. Introduction

The accurate and sensitive detection of H_2_O_2_ content is of great significance in clinical diagnostics, food safety, environmental monitoring and other fields [1]. Therefore, more detection techniques such as titration analysis [2], spectrophotometry [3], chemiluminescence [4], and electrochemical methods [5] are used to analyze H_2_O_2_. Among these methods, electrochemical methods are widely used and are widely reported. It is generally believed that ampere-based electrochemical biosensors based on fixed horseradish peroxidase (HRP) are the most effective and commonly used methods for detecting H_2_O_2_. However, the denaturation and shedding of surface enzymes on the sensor electrode of immobilized enzymes and other bioprotein molecules has been a major obstacle to the application of enzyme sensors, especially commercial applications, because it directly affects the sensitivity, stability and repeatability of the sensor. It is still a key issue in enzyme sensors that has to be overcome so far. At present, the immobilization of an inorganic material with a direct electrocatalytic activity and a very stable nature to the surface of the electrode to construct a non-enzymatic sensor can effectively overcome the problems caused by the enzyme-based sensor, since the material with stable properties does not undergo any denaturation. Some non-enzymatic sensors for detecting H_2_O_2_ have been reported in the literature. For example, metals like silver platinum, gold or their combination [6,7,8,9,10,11]; WS_2_ [12] and electrogenerated ferrites or their combination with carbon nanomaterials [13,14] have been successfully used for the analytical determination of hydrogen peroxide.

Porous carbon materials (PC) are widely used in gas adsorption separation, water and air purification, catalyst carriers, chromatography, electrochemical double layer capacitors and fuel cells [15,16], due to their high specific surface area, pore volume, catalytic properties, good mechanical stability, etc. In addition, studies have found that natural forms of porous carbon play an important role in both electrocatalysis and electron transport processes. However, due to the limited research in this area, the electrocatalytic applications of these natural low-cost porous carbon materials are greatly limited. In this work, we prepared a non-enzymatic sensor of EPC/CHIT/GCE by using chitosan (CHIT) with good stability and excellent film forming properties as a film-forming agent to fix the eucalyptus biomorphic porous carbon material (EPC) to the surface of the glassy carbon electrode (GCE). The non-enzymatic sensors constructed by EPC are characterized by low cost, simplicity, ease of use, and long-term stability. To the best of our knowledge, the use of naturally occurring porous carbon as a catalyst for non-enzymatic sensors has been reported for the first time. It is believed that it will have a broader application in the field of analytical chemistry and electronic device research.

## 2. Materials and Methods

### 2.1. Reagents and Instruments

The original ecological eucalyptus was a carbonized material for the preparation of biomorphic porous carbon. Chitosan (CHIT, MW~ 1 × 10^6^, >85% deacetylated) and hydroquinone were purchased from Sigma (Shanghai, China). Hydrogen peroxide (H_2_O_2_, 30% V/V aqueous solution) was obtained from Shanghai Biochemical Reagent Company (Shanghai, China). Other analytical grade chemicals were purchased from Beijing Chemical Reagent Company (Beijing, China) and did not require further purification. Phosphate buffer solution (PBS, 0.067 M) was prepared by using K_2_HPO_4_ and NaH_2_PO_4_ standard solutions and adjusting the pH to 7.0. All solutions were prepared with deionized water.

The electrochemical experiment was carried out on a CHI660B electrochemical workstation (Shanghai Chenhua Instrument Co., Ltd., Shanghai, China) under normal temperature conditions, and a three-electrode system was used: A platinum electrode as a counter electrode; a KCl-saturated Ag/AgCl electrode as a reference electrode; and a 3 mm diameter glassy carbon electrode as the working electrode. A scanning electron microscope (SEM) image was obtained by JEM-6700F (JEOL, Tokyo, Japan). Fourier transform infrared spectroscopy (FT-IR) was obtained by using KBr as a compression background on a WQF-410 infrared spectrometer (Beijing Second Optical Instrument Co., Ltd., BeiJing, China). The XRD of EPC was characterized by the Siemens D5000 (München, Germany).

### 2.2. Preparation of EPC Materials

The processed eucalyptus block with a diameter of 5–10 mm was first dried at 120 °C for 48 h and then heated to 600 °C at a heating rate of 2 °C/min and maintained at 600 °C 3 h to completely decompose the polyaromatic complex into carbon; The temperature was raised to 1200 °C at a temperature rise rate of 5 °C/min, and the temperature was maintained for 4 h to produce crack-free carbon; finally, the eucalyptus porous carbon (EPC) material was obtained by naturally cooling to room temperature. It is to be noted that the entire process was carried out under inert gas protection conditions.

### 2.3. Preparation of EPC/CHIT/GCE Electrochemical Sensors

Prior to electrode modification, the glassy carbon electrode was subjected to a series of grinding processes on alumina powders of different particle sizes, ultrasonically washed with ethanol and water, and finally blown dry with nitrogen. After the electrode was processed, the EPC was first ultrasonically dispersed with water to obtain a uniform dispersion with a concentration of 1 mg/mL. After thoroughly mixing the 0.5%-wt CHIT solution with the EPC suspension, 5 μL of the above mixed droplets were taken out and added to the surface of the glassy carbon electrode, and naturally dried to obtain EPC/CHIT/GCE. The sensor was placed in PBS solution and stored at room temperature. As a control experiment, a bare glassy carbon electrode and a CHIT modified glassy carbon electrode (CHIT/GCE) were prepared by a method similar to that described above.

## 3. Results and Discussion

### 3.1. Characterization of EPC Materials

**Figure 1:**
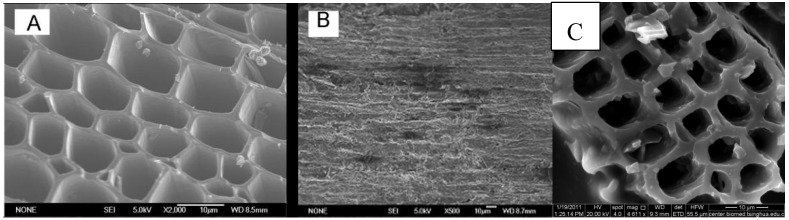
SEM images of EPC and EPC/CHIT/GCE: (**A**,**B**) perpendicular and parallel to the axial direction of EPC; (**C**) EPC/CHIT/GCE.

The eucalyptus was composed of dense cells with small pores, and the interstitial structure was uniform and dense. The carbonized EPC still maintained this regular and orderly structure. Figure 1 is a Scanning Electron Microscope (SEM) image of the EPC and EPC/CHIT/GCE. From Figure 1A we can see that the EPC has a honeycomb shape and is a hollow channel structure with different diameters. This structure is derived from the structure of the natural form of eucalyptus. The black part of the figure is the cell cavity of the eucalyptus, while the gray part is the cell wall of the eucalyptus. Most of the holes have an elliptical topography and are connected to each other through the wall of the hole. These cells are parallel to the axial direction of the elm texture structure (Figure 1B). The anisotropic microstructure of EPC is different from other natural porous materials (such as zeolite, diatomaceous earth, etc.), and this dense one-way natural pore structure greatly increases its specific surface area. The structural morphology of the EPC/CHIT/GCE on the electrode surface was clearly seen by scanning electron microscopy (Figure 1C). The material maintains its unique three-dimensional structure even on the electrode surface, which is porous. The array layout hardly changed.

**Figure 2:**
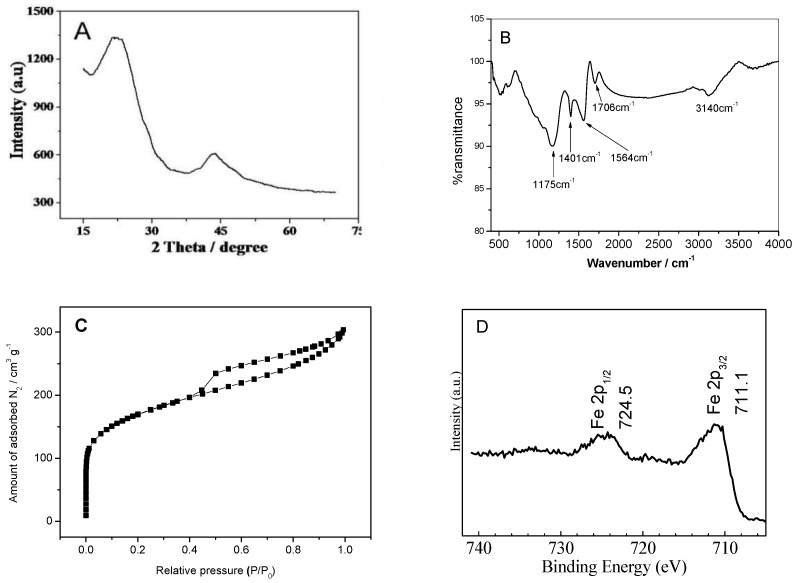
Characterization of EPC materials: (**A**) XRD; (**B**) FT-IR; (**C**) BET and (**D**) XPS spectroscopy.

As can be seen from the XRD of the EPC (Figure 2A), there are two broad peaks appearing at 24° and 44°, respectively assigned to the (002) peak (101) peak of amorphous carbon. These two characteristic peaks indicate that the material after carbonization is amorphous carbon [17]. It was found by FT-IR absorption spectroscopy (Figure 2B) that the EPC contained more functional groups. The main absorption bands of the synthetic materials in the range of 400–4000 cm^−1^ were 3135, 1706, 1564 and 1175 cm^−1^, which are attributed to the stretching vibration of hydroxyl groups, the stretching vibration of carbonyl groups, C-O (hydroxyl, ester, or ether) stretching and O-H bending vibration, respectively [18]. The N_2_ adsorption-desorption isotherms and X-ray photoelectron spectroscopy of the EPC materials are shown in Figure 2C,D. The nitrogen adsorption-desorption isotherms show an obvious hysteresis loop, which indicates that the EPC material is porous. The surface area of the EPC materials was 598.1 m^2^·g^−1^, calculated using the Brunauer–Emmett–Teller (BET) equation. The X-ray photoelectron spectroscopy (XPS) showed that the EPC material contained a small amount of iron in addition to carbon and oxygen (Figure 2D).

### 3.2. Cyclic Voltammetry

**Figure 3:**
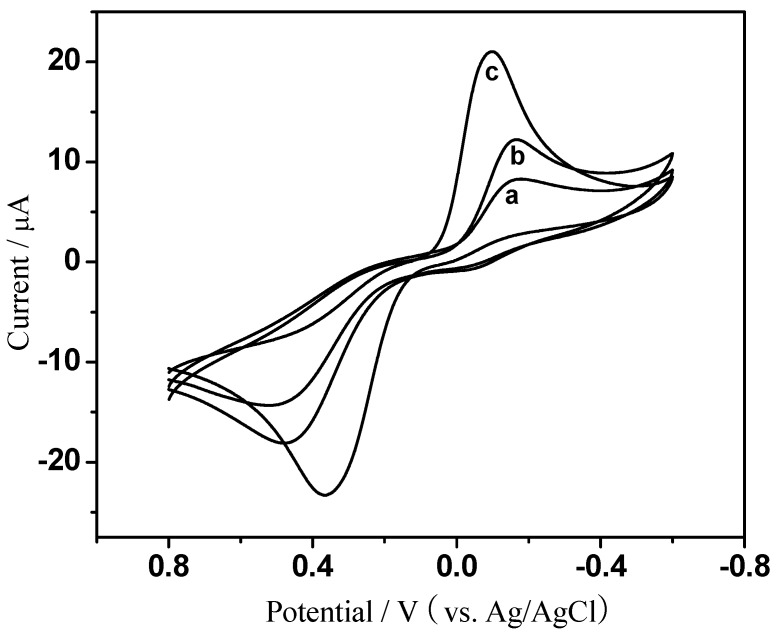
Cyclic voltammetry curves for different modified electrodes: (a) CHIT/GCE; (b) bare glassy carbon electrode; (c) EPC/CHIT/GCE. The buffer solution was N_2_ saturated 0.067 M PBS (pH 7, containing 1 mM hydroquinone) at a scan rate of 100 mV·s^−1^.

**Figure 4:**
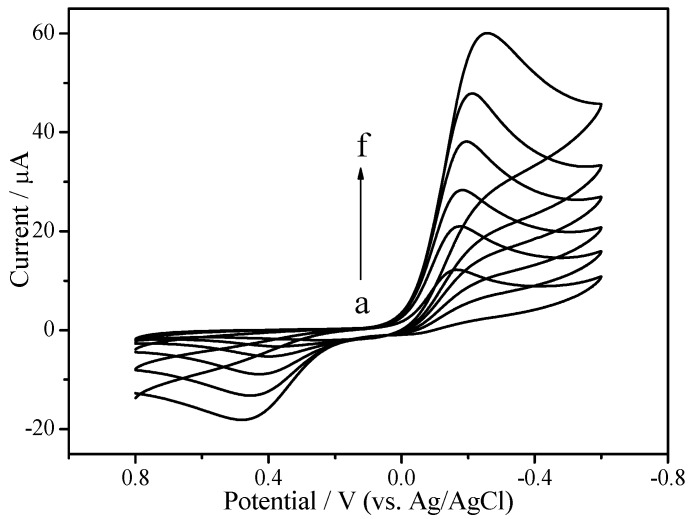
Cyclic voltammetry curves of EPC/CHIT/GCE with different concentrations of H_2_O_2_: (a) 0 mM; (b) 1 mM; (c) 1.5 mM; (d) 2 mM; (e) 2.5 mM and (f) 5 mM. The buffer solution was N_2_ saturated 0.067 M PBS (pH 7, containing 1 mM hydroquinone) at a scan rate of 100 mV·s^−1^.

The properties of EPC that promoted electron transfer were first investigated in PBS containing 1 mM hydroquinone. Figure 3 summarizes the electron transfer characteristics of different modified electrodes. Under the condition of −0.2 V operating potential with Ag/AgCl and no hydrogen peroxide, a pair of hydroquinone redox peaks appeared in different modified electrodes. It is apparent from the figure that the redox peak of CHIT/GCE (curve a, Figure 3) was smaller than the bare GCE (curve b, Figure 3), which indicates that CHIT itself may hinder electron transport. However, EPC/CHIT/GCE had a pair of redox peaks with significantly larger current values and a smaller peak difference (curve c, Figure 3). The electrocatalytic activity of EPC/CHIT/GCE for H_2_O_2_ was further investigated by cyclic voltammetry. In the absence of hydrogen peroxide, a typical redox characteristic peak of hydroquinone (curve a, Figure 4) occurred when a certain concentration of hydrogen peroxide was added to the PBS at about −0.2 V (vs. Ag/AgCl) showed a significant increase in the reduction peak current, accompanied by a decrease in the oxidation peak current, and as the H_2_O_2_ concentration in the system increased, the reduction peak current also increased (curve b to f, Figure 4). The experimental results show that EPC can be used as an excellent non-enzymatic catalyst for the construction of non-enzyme electrochemical sensors for detecting H_2_O_2_. It was fully demonstrated that EPC can promote the transfer of electrons between the catalytically active center and the electrode surface. The catalytically active center may be present in the three-dimensional biomorphological structure of the pore structure or the surface of the material.

**Figure 5:**
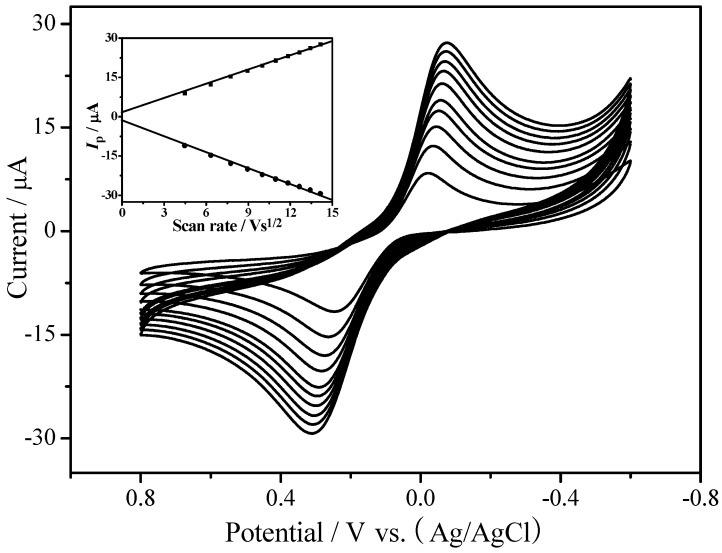
Cyclic voltammetry curves of EPC/CHIT/GCE at different scan rates: from inside to outside at 20, 40, 60, 80, 100, 120, 140, 160, 180, 200 mV·s^−1^; the input is the anode and cathode peak current and the scan rate square root curve. The buffer solution was nitrogen-saturated 0.067 M PBS (pH 7, containing 1 mM hydroquinone).

Figure 5 shows the electrochemical response of EPC/CHIT/GCE at different scan speeds. The sweep rate was changed from 20 mV·s^−1^ to 200 mV·s^−1^, and the redox peak current value was found to be proportional to the square root of the scan speed (Figure 5), indicating that the electrochemical process was controlled by diffusion. The peak-to-peak potential difference between the cyclic volt-ampere of the sweep speed from 40 mV·s^−1^ to 180 mV·s^−1^ showed that the average electron transfer rate parameter *k_s_* of EPC fixed in CHIT was 1.06 s^−1^. This value was calculated according to the Laviron model using the formula *k_s_ = mnFv/RT*. In the formula, *n* represents the electron transfer number; *F* represents the Faraday constant; *m* is the peak-to-peak potential difference parameter; *v* is the scanning speed; *R* is the gas constant; and *T* is the temperature, respectively [19]. Here, *T* = 298 K, *n* = 1. The *k_s_* in this study were smaller than the reported biosensors using enzymes as catalysts [20,21], which further demonstrates that EPC can promote electron transfer.

### 3.3. Sensor Response Characterization

**Figure 6:**
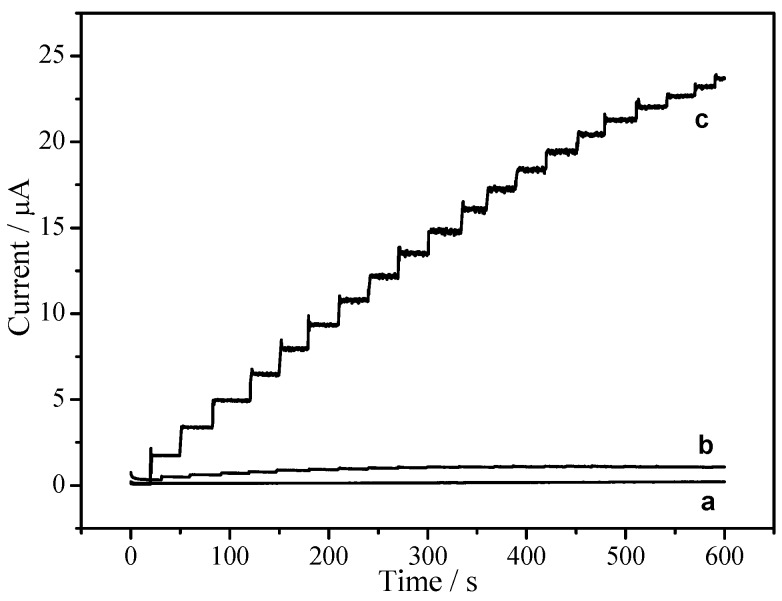
Time-current curve of continuous addition of 0.1 mM H_2_O_2_ to different modified electrodes: (a) CHIT/GCE; (b) bare GCE; (c) EPC/CHIT/GCE. The buffer solution was N_2_ saturated 0.067 M PBS (pH 7, containing 1 mM hydroquinone), and the operating potential was −0.2 V vs. Ag/AgCl (sat. KCl).

In order to compare the electrocatalytic properties of EPC/CHIT/GCE with CHIT/GCE and bare GCE, the catalytic performance of three modified electrodes were investigated by the electrocatalytic reduction of H_2_O_2_. Figure 6 is a current-time (it) curve for three modified electrodes. When adding 10 μL of 0.1 M H_2_O_2_ to 10 mL of PBS containing 1 mM hydroquinone, CHIT/GCE and bare GCE had almost no apparent catalytic response (see lines a and b in Figure 6), whereas EPC/CHIT/GCE (see line c in Figure 6) showed a significant catalytic response to different concentrations of H_2_O_2_ with a typical current-time response curve. Experiments show that the electrochemical sensor had a fast and sensitive response to H_2_O_2_.

With a signal-to-noise ratio of 3 and a correlation coefficient of 0.998 (*n* = 15), the linear detection range for H_2_O_2_ ranged from 15 μM to 1.6 mM with a minimum detection limit of 3.7 μM and sensitivity theory for EPC/CHIT/GCE. The calculated value was 204.5 μA·mM^−1^·cm^−2^. Table 1 summarizes the analytical parameters for the most relevant non-enzymatic hydrogen peroxide sensors based on the use of different metallic nanoparticles reported since 2013. The comparison allowed us to conclude that our sensor presented a comparable [22,23,24,25] linear range and sensitivity to most of the sensors. However, our sensor presented the great advantage of easy preparation and no precious metals.

The Michaelis constant (*K*_m_) is a kinetic parameter for studying the binding catalysis of an enzyme or catalyst to a substrate. In general, a smaller *K*_m_ indicates a higher affinity of the enzyme or catalyst to the substrate. This value can be calculated using the Lineweaver-Burk equation (Equation (1)) [26].

(1) $$\frac{1}{I_{\mathrm{ss}}}=\frac{1}{I_{\max}}+\frac{K_{m}}{I_{\max}}\cdot \frac{1}{C}$$

**Figure 7:**
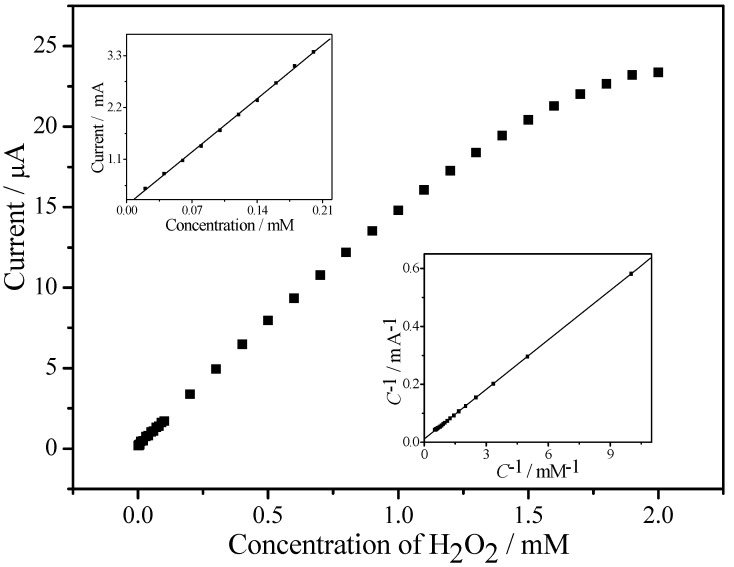
Current-concentration curve of EPC/CHIT/GCE response to H_2_O_2_: The upper inset is the current-concentration curve for low-concentration H_2_O_2_; the lower inset is the Lineweaver-Burk curve for the sensor.

In this equation, *I*_ss_ is the steady state current value after substrate addition; *I*_max_ is the maximum current at substrate concentration saturation; and *C* is the substrate concentration. In this work, the calculated value was 3.67 mM (see insert diagram (bottom), Figure 7). This value was lower than the 8.01 mM based on the HRP-ZrO_2_ nanocomposite sensor [27] and much higher than the 41 mM based on the Nafion-Hb-CNT sensor [26]. Comparison of the surface results, especially with enzyme sensing, showed that EPC had higher enzymatic activity and had a good affinity for H_2_O_2_.

### 3.4. Sensor Selectivity, Reproducibility and Stability

Several possible interfering substances were selected to examine the selectivity of the sensor. The experiment was to compare the response current of the sensor to 0.1 mM H_2_O_2_ in the presence or absence of 1.0 mM interfering substances. The experimental results (Table 2) indicate that glucose, ethanol, oxalic acid, and uric acid do not substantially affect the sensor’s determination of H_2_O_2_. 

To further determine the performance of this non-enzymatic sensor, we investigated its operational repeatability and long-term stability. The operational stability of the 2 mM H_2_O_2_ electrocatalyst for 7 h was investigated by the same EPC/CHIT/GCE, and the results showed no significant change or difference. The standard deviation of H_2_O_2_ at the same concentration for ten consecutive times was only 0.8%, indicating that the sensor has good repeatability. In order to evaluate the repeatability between the different modified electrodes, the standard deviation of the response of the five separately prepared electrodes to the same concentration of H_2_O_2_ under the same conditions was only 2.3%, indicating that the electrodes still had high repeatability, which further explains the sensor with high practicality. The long-term stability was also systematically studied, and the same sensor was used to detect the same concentration of H_2_O_2_ every few days. After one month of research, the data showed no significant change, which means that the sensor had high long-term stability. The most critical reason for achieving such high repeatability and stability is that the EPC does not undergo denaturation or reduced activity like other proteins or enzymes, and the physicochemical properties of the material are extremely stable. 

### 3.5. Possible Response Mechanism

Based on the above experimental results, it is speculated that the possible mechanism of the non-enzymatic sensor’s response to hydrogen peroxide is as follows:

eucalyptus porous carbon (Fe^2+^) + H_2_O_2_ + H^+^ → eucalyptus porous carbon (Fe^3+^) + H_2_O

eucalyptus porous carbon (Fe^3+^) + H_2_Q → eucalyptus porous carbon (Fe^2+^) + Q + H^+^

Among them, H_2_Q and BQ represent the electron mediator hydroquinone and its oxidized form (p-benzoquinone), respectively. Under the action of hydroquinone, two electron transfers occurred first, H_2_O_2_ was reduced to water, and the divalent iron ions in the porous carbon of eucalyptus were oxidized to ferric ions, while ferric ions in the eucalyptus porous carbon were reduced to divalent iron ions by hydroquinone. Subsequently, the p-benzoquinone rapidly acquired two electrons from the electrode and was reduced to hydroquinone. The peak current in the system is therefore related to the concentration of H_2_O_2_ in the solution.

## 4. Conclusions

In this paper, a porous carbon material with biological morphology was prepared by a simple and effective carbonization technique. The prepared EPC had high electrocatalytic activity and affinity for H_2_O_2_ with the aid of hydroquinone. The non-enzyme electrochemical sensors prepared using the ordered porous material had high sensitivity, high accuracy, high repeatability and stability. In addition, EPC provides researchers with new examples of non-enzymatic electrochemical sensors using natural structural carbon materials. It is believed that this type of naturally-ordered porous carbon material will have potential research and application value in the field of catalysis and analysis.

## Figures and Tables

**Table 1:** Analytical characteristics of different non-enzymatic hydrogen peroxide sensors.

Platform	Detection Limit (μM)	Sensitivity (μA·mM^−1^·cm^−2^)	Linear Range (μM)	Reference
Pt/PG/GCE	<0.50	341.14	1–1477	[22]
Pt-IL-pGR-GCE	0.42	942.15	10–4000	[23]
Au@C@Pt/GCE	0.13	144.7	9–1860	[24]
rGO/FeNPs nanocomposite	0.06	208.5	0.1–2150	[25]
EPC/CHIT/GCE	3.7	204.5	15–1600	This work

**Table 2:** Interference experiments of EPC/CHIT/GCE electrodes.

Interference	Ratio of Current Values ^a^
glucose	1.00
ethanol	1.00
oxalic acid	1.02
uric acid	0.98

^a^ is the current value of 1 mM interference and 0.1 mM hydrogen peroxide and comparison with only 0.1 mM H_2_O_2_.
